# Fractional Gluconeogenesis: A Biomarker of Dietary Energy Adequacy in a Rat Brain Injury Model

**DOI:** 10.3390/metabo12121163

**Published:** 2022-11-23

**Authors:** Casey C. Curl, Anika Kumar, Austin J. Peck, Jose A. Arevalo, Allison Gleason, Robert G. Leija, Adam D. Osmond, Justin J. Duong, Benjamin F. Miller, Michael A. Horning, George A. Brooks

**Affiliations:** 1 Department of Integrative Biology, University of California at Berkeley, Berkeley, CA 94720, USA; 2 Aging and Metabolism Research Program, Oklahoma Medical Research Foundation, Oklahoma City, OK 73104, USA; 3 Oklahoma City Veteran Affairs Medical Center, Oklahoma City, OK 73104, USA

**Keywords:** TBI, tracer, glycemia, D_2_O, metabolism, fasting

## Abstract

Patients treated for traumatic brain injury (TBI) are in metabolic crises because of the trauma and underfeeding. We utilized fractional gluconeogenesis (fGNG) to assess nutritional adequacy in ad libitum-fed and calorically-restricted rats following TBI. Male Sprague–Dawley individually housed rats 49 days of age were randomly assigned into four groups: ad libitum (AL) fed control (AL-Con, sham), AL plus TBI (AL+TBI), caloric restriction (CR) control (CR-Con, sham), and CR plus TBI (CR+TBI). From days 1–7 animals were given AL access to food and water containing 6% deuterium oxide (D_2_O). On day 8, a pre-intervention blood sample was drawn from each animal, and TBI, sham injury, and CR protocols were initiated. On day 22, the animals were euthanized, and blood was collected to measure fGNG. Pre-intervention, there was no significant difference in fGNG among groups (*p* ≥ 0.05). There was a significant increase in fGNG due to caloric restriction, independent of TBI (*p* ≤ 0.05). In addition, fGNG may provide a real-time, personalized biomarker for assessing patient dietary caloric needs.

## 1. Introduction

In 2019, traumatic brain injuries (TBI) resulted in 223,135 hospitalizations, and 60,611 deaths [1]. TBI recovery can span days to weeks, or longer [2]. Practitioners in neurological intensive care units (neuro ICU) are responsible for the caloric needs of TBI patients. Such needs are technically difficult and of low priority given other, urgent exigencies. Hence, TBI patients typically deal with both standard of care (SOC) underfeeding [3,4] and injury provoked metabolic crises [4,5,6,7,8,9,10,11,12,13]. The interplay between dietary energy deficit and recovery from TBI is relatively unstudied.

In healthy non-diabetic humans, fractional gluconeogenesis (fGNG) ranges between 40% and 60% following an overnight fast [14], and rises to 90% with prolonged fasting [15,16]. Previously, it has been observed that neuro ICU patients suffering from TBI had increased fGNG compared to non-injured control subjects [6,12]. Because of the inadequacy of Harris–Benedict (H–B) equations for predicting the energy needs of individual patients [3,4], as well as difficulties in using indirect calorimetry for assessing metabolic rate, or assessing nitrogen balance in the critical care setting, clinicians have described the need for a biomarker of dietary energy adequacy in TBI patients, among others [17]. In the clinical setting, measurements of fGNG could provide a biomarker to avoid under- or overfeeding of trauma patients [6,18]. While it is unknown why fGNG raises quickly in TBI patients, they typically face dual challenges of injury-induced metabolic crises and undernutrition [6,19].

The purpose of this study was to determine if increased endogenous glucose production via fGNG is due to TBI, dietary energy inadequacy from caloric restriction (CR), or a combination of the two stressors. We hypothesized that TBI in conjunction with CR would result in a greater increase in fGNG than CR alone. We found that caloric restriction raised fGNG independent of TBI.

## 2. Experimental Design

**Figure 1:**
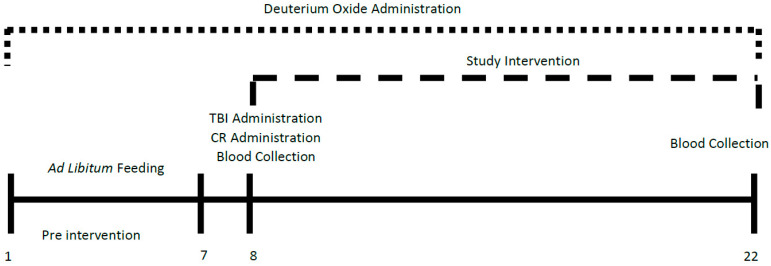
An experimental schematic of the study. Animals were housed individually, and on day 1 we provided 6% D_2_O drinking water that was maintained for the entirety of the study (21 days). Days 1–7 were considered pre-study where the animals were given AL access to food; food intake and water intake were measured daily. On day 8 the rats were randomized into the study groups: Ad libitum (AL) fed control (AL-Con, sham), AL plus TBI (AL+TBI), CR control (CR-Con, sham), and CR plus TBI (CR+TBI). Following a blood draw, the animals received a TBI or a sham intervention. Following the TBI or sham intervention, half rations were provided to the animals selected for CR treatment. From days 9–21, the animals were allowed to recover in their cages. On day 22 the animals were euthanized, and blood was collected.

Procedures conducted on the animals were approved by the University of California, Berkeley Animal Care and Use Committee (2018-08-11312). Male Sprague–Dawley rats were purchased from Charles River, USA (Wilmington, MA) at 49 days of age simulate an adolescent, not completely developed brain, and housed individually. Cages were maintained at a constant temperature and humidity with a 12-h light–dark cycle (Light: 7:00 a.m. to 7:00 p.m.). Figure 1 shows the schematic for our study design. The animals were given free access to 6% deuterium oxide (D_2_O, heavy or labeled water) for the entirety of the study, and ad libitum (AL) access to standard chow mix (PicoLab Rodent Diet 20: 62:13:25% carbohydrate, fat, and protein) for the first 7 days.

On day 8, a free rotation closed-head traumatic brain injury (TBI) [20] was induced. Following TBI, animals were randomly assigned to four groups based on injury and nutritional interventions: 1. Sham injury control with AL feeding (AL-Con), 2. TBI and AL feeding (AL+TBI), 3. TBI and half ration caloric restriction (CR+TBI), 4. Sham injury and caloric restriction (CR-CON). Individual rat weights were recorded periodically throughout the study (Table 1). The half-ration, CR treatment, was designed to mimic human patient treatment in a Neuro ICU [6,18]. For the first 7 days, food trays were weighed to calculate half ration for the groups that received CR (Table 2). Then, the average of each rat’s daily food consumption prior to TBI was calculated and rounded up the nearest half gram and divided by two to determine the CR group daily allotment. Per the NIH Subcommittee on Laboratory Animal Nutrition, the AL food consumption of a Sprague–Dawley rat is considered to be the food necessary to meet the animals’ daily energy requirements [21], in other words, caloric adequacy. Thus, by limiting the animals’ food intake by 50% we caused caloric deficits in the CR groups.

Food was removed from animal cages 24 h before euthanasia while 6% D_2_O remained continuously available for the animals. Animals were euthanized on day 22 via carbon dioxide asphyxiation followed by decapitation. Subsequently, blood was collected in EDTA tubes and spun at 3000 g for 18 min to separate the plasma which was stored at −20 °C until analysis

### 2.1. Traumatic Brain Injury Model

Prior to injury, the animals were anesthetized using 3.5% isoflurane atomized in oxygen at a low rate of 1 L/min for approximately 15 min. If breathing rate remained elevated or toe-pinch reflex was present, the animals continued under anesthesia for an additional minute, or until the toe-pinch reflex was no longer detected. Animals were then quickly moved to a perforated foil platform 8 cm above a 7.6 cm thick medium-density foam pad in a prone position. The bolt was positioned on the rat’s head along the midline and aligned with the ears to target between the lambda and bregma skull landmarks. After confirming the toe-pinch reflex had not been regained, a 450 g weight was dropped from 135 cm onto a 3 cm of bolt throw. Sham animals underwent the same course of anesthesia and placement on the apparatus with no weight drop. Immediately post-impact, the animals were returned to a clean cage in the supine position and observed.

### 2.2. Labeled Water and Body Water Enrichment Analysis

Animals were given AL access to 6% D_2_O drinking water for the entirety of the study (days 1–22). There were no bolus D_2_O injections administered due to the fast nature at which D_2_O incorporates into body water pools [22]. Assessment of body water enrichment was as described by Miller et al. [22,23]. Briefly, 120 μL of plasma was placed into the cap of inverted screw-capped tubes and placed in a heat block for overnight distillation at 80 °C. Distilled samples were diluted 1:300 in doubly-distilled (dd) H_2_O and analyzed on a liquid water isotope analyzer (Los Gatos Research, Los Gatos, CA, USA) against a standard curve prepared with samples containing different percentages of D_2_O.

### 2.3. Fractional Gluconeogenesis Measurement

A glucose penta-acetate derivative was used to measure deuterium incorporation into the glucose molecule via GNG. For this, 25 μL of plasma was placed into a 1.5 mL Eppendorf tube containing 50 μL of ethanol. A liquid-to-liquid ethanol extraction was performed and the organic layer containing glucose was extracted and transferred to a 2 mL glass vial and dried under nitrogen (N_2_) gas. The dried glucose was derivatized using 100 μL of a 2:1 mixture of acetic anhydride and pyridine. The GC vial was sealed and heated at 60 °C for 20 min. After 20 min, the sample was dried under N_2_ and then reconstituted in 500 μL of ethyl acetate.

Isotopic enrichments of glucose penta-acetate derivative were measured on an Agilent 6890/5973 Gas Chromatograph–Mass Spectrometer (GC-MS) utilizing positive chemical isolation (PCI) and selected ion monitoring (SIM) on a DB-17 GC column. The starting oven temperature was 110 °C, increased by 35 °C every minute till 225 °C, and held for 5 min. The mass-to-charge ratios (*m/z*) of 169 and 170 were monitored for the glucose penta-acetate derivative.

To calculate fGNG, body water enrichment was used in conjunction with SIM measurements of *m/z* 169 and 170 of the glucose penta-acetate derivative, as previously described [24]. Briefly, the 169 fragment of the glucose penta-acetate derivative contains six hydrogen atoms at carbons 1, 3, 4, 5, and 6. Unlike other fragments of the glucose derivative, the 169 fragment lacked the hydrogen on carbon 2 (C2), which is proportional to the total glucose production in the body due to the required glucose-6-phosphate to fructose-6-phosphate isomerization that takes place at that position [24]. Deuterium enrichment (*m/z* 170) at all carbon sites excluding the hydrogen/deuterium on C2 is most indicative of gluconeogenesis as they represent new glucose production solely from the gluconeogenic pathway. Thus, the average enrichment of the glucose penta-acetate fragment was calculated using the equation below by the SIM of *m/z* 170/169 and subsequently dividing by 6 because of the six possible sites on the glucose molecule at which a hydrogen atom could potentially exchange with a deuterium atom (Equation (1)).

**169* fragment enrichment = [(M + *1*) _(m/z *170*_ /(M) _(m/z *169*)_]/*6** (1)

To calculate fGNG, the average 170/169 glucose penta-acetate fragment enrichment was divided by the body water enrichment (Equation (2)).

*fGNG = *169* fragment enrichment/Body Water Enrichment* (2)

### 2.4. Blood Glucose Analyses

Whole blood was collected in EDTA tubes. Aliquots of these samples were added 1:2 to tubes containing 8% perchloric acid (200:400 μL) to stop metabolism, vortexed, and then immediately centrifuged at 2000× *g* for 10 min. Perchloric acid extracts were neutralized with 2 N NaOH. Glucose concentrations were then measured with a hexokinase enzymatic solution from Thermo Fisher Scientific (Waltham, MA, USA).

### 2.5. Kinematic and Behavioral Analyses

To measure the severity of the TBI impact on the animals we performed a multitude of modified neurological severity score (mNSS) tests [25]. We utilized open field, beam-walk, and inverted wire mesh holds to measure cognitive impairments due to TBI. Behavioral analyses were performed at six time points: 30 min (min) pre-TBI, 30 min, 24 h, 48 h, 72 h, and 10 days post-TBI. A light aversion assay [26] was administered to test the animals’ sensitivity to light 13 days post-injury.

### 2.6. Statistical Analyses

All statistical analyses were run utilizing SPSS (IBM SPSS Statistics for Macintosh, Version 27.0 Armonk, NY, USA: IBM Corp). To find the difference in food consumption between our pre-TBI, 24 h post-intervention, and final food consumption (13 days post-intervention), we performed a two-way ANOVA. Significance was set at *p* ≤ 0.05 and confirmed with Tukey’s post hoc test. Similarly, we used a two-way ANOVA to measure the differences in weight change between each group from pre-TBI, 24 h post-intervention, and final weight (13 days post-intervention) measurements; significance was set at *p* ≤ 0.05 and confirmed with a Tukey’s post hoc test. Final glucose concentrations were measured using a two-way ANOVA to find group differences, and significance was set at *p* ≤ 0.05. We performed an independent *t*-test to measure the difference in fGNG between the AL and CR groups, as well as an independent *t*-test to measure the difference in fGNG between the control and TBI groups. Finally, we utilized a two-way ANOVA using feeding status and TBI status as the independent variables to find an interaction effect between TBI and CR, and fGNG. Significance was set at *p* ≤ 0.05 and confirmed with Tukey’s post hoc test. We performed a two-way ANOVA to find group differences on all behavioral testing. For all behavioral testing, we conducted a two-way ANOVA using TBI and feeding status as the independent variables. Significance was set at *p* ≤ 0.05 and confirmed with Tukey’s post hoc test.

## 3. Results

### 3.1. TBI Leads to Alterations in Light Sensitivity 13 Days Post-Injury

Of the numerous state-of-the-art pre- and post-TBI behavioral assessments performed, injured animals showed behavioral change only due to light exposure via the light aversion assay after recovering from TBI; that is, animals spending less time in the light field at 500 lux (TBI: 35+ 15 s vs. Con: 45 ± 15 s *p* ≤ 0.05) and 750 lux (TBI: 31.2 ± 13 s vs. Con: 42 ±10 s *p* ≤ 0.05) than the control animals. By contrast, there were no differences in inverted wire mesh hold time (TBI: 7 ± 3.1 s, AL 6 ± 2.4 s *p* ≥ 0.05), total distance traveled in the open field (TBI: 400 ± 254.7 cm vs. Con 378 ± 266.9 cm *p* ≥ 0.05), and time to reach the end of the walking beam (TBI: 5 ± 2.4 s vs. Con: 5.2 ± 2 s *p* ≥ 0.05) between the TBI and Con groups. Hence, it appears that the free rotation, closed-head traumatic brain injury method we used produced a mild TBI.

### 3.2. TBI and CR Significantly Alter Body Weight and Voluntary Food Consumption Post-TBI

On the day of the intervention (day 8), there were no weight differences between AL-Con, AL+TBI, and CR+TBI groups. Despite the randomization of treatment, the CR-Con group had a significantly higher starting weight (*p* ≤ 0.05) (Table 1). Subsequently, 24 h post-TBI there was a significant decrease in weight in both TBI groups, (*p* ≤ 0.05), with no changes in the CR-Con group, and a significant increase in weight in the AL-Con group (*p* ≤ 0.05). Thirteen days post-intervention there were significant weight gains by the AL groups (AL-CON and AL+TBI; *p* ≤ 0.001). There was no difference in the weight change between the AL groups (*p* ≥ 0.60). Additionally, 13 days post-intervention there was a significant decrease in weight in the CR groups (*p* ≥ 0.001), with no difference in the amount of weight loss between the two groups (*p* ≥ 0.37). There was a significant effect of caloric restriction on weight change (AL: 37.11 ± 17.61 g vs. CR: −20.36 ± 12.76 g; *p* ≤ 0.001), with no significant effect of TBI on weight change (CON: 5.77 ± 31.36 g vs. TBI: 5.27 ± 35.49 g; *p* ≥ 0.95). We were not able to find an interaction between CR and TBI. Thus, caloric restriction, not TBI, has a significant effect on weight changes post-TBI. Our data show that animals on a CR diet deviated negatively from the natural growth projection of animals their age, while AL animals had a similar growth progression to age-matched animals [21].

Food consumption behaviors are reported in Table 2. There were no differences in food consumption between groups prior to the intervention (*p* ≥ 0.23). There was a significant decrease in food consumption from pre- to 24 h post-intervention in the AL+TBI group (*p* ≤ 0.005), and the two caloric restriction groups (CR-Con and CR+TBI, *p* ≤ 0.005), with no change in food consumption in the AL-Con group (Table 2). At the conclusion of the study, 13 days post-intervention, as expected via study design there was a significant decrease in food consumption in the CR groups (*p* ≤ 0.001). However, there was no difference in food consumption between AL-Con and AL+TBI (Table 2). Thus, the CR+TBI treatment group mimicked what was observed in a state-of-the-art neural ICU.

### 3.3. Caloric Restriction Increases Fractional Production of Endogenous Glucose via Gluconeogenesis with No Effect on Blood Glucose Concentrations

**Figure 2:**
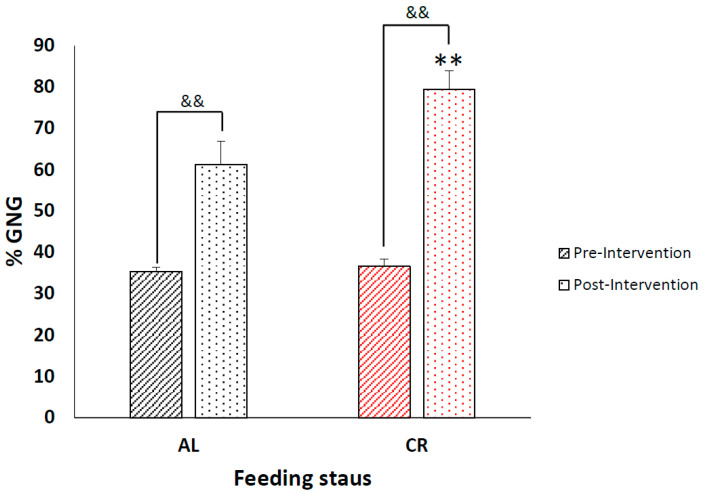
Caloric restriction significantly increased endogenous glucose production. Percent fGNG pre-intervention and 13 days post-caloric restriction in AL vs. CR groups. Black angled lines indicate AL fed pre-intervention fGNG, black dotted columns indicate AL fGNG 13 days post-intervention. The red angled lined column indicates CR fed pre-intervention fGNG, Red dotted column represents CR fGNG 13 days post-intervention. ** significantly different from AL group *p* ≤ 0.001, && significantly different from pre-intervention ≤0.001.

**Figure 3:**
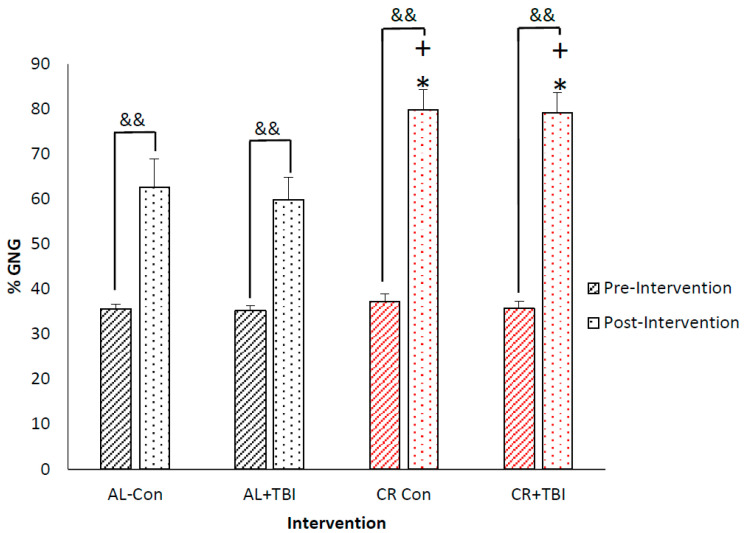
Caloric restriction significantly increased endogenous glucose production independent of TBI. Percent fGNG pre-intervention and 13 days post-caloric restriction in AL-Con, AL-TBI, CR-Con, and CR-TBI groups. Black angled lines indicate AL fed pre-intervention fGNG, black dotted columns indicate AL fGNG 13 days post-intervention. The red angled lined column indicates CR fed pre-intervention fGNG; the red dotted column represents CR fGNG 13 days post-intervention. * *p*≤ 0.001 from AL-Con, + *p* ≤ 0.001 from AL+TBI, && significantly different from pre-intervention ≤0.001.

The average pre-intervention fGNG for the AL (35.40 ± 1.10%) and CR (36.62 ± 1.71%) groups were not significantly different (*p* ≥ 0.55). At the conclusion of the study, 13 days post-intervention, there was a significant increase in fGNG with CR (AL: 61.25 ± 5.68% vs. CR: 79.46 ± 4.47%, *p* ≤ 0.001) (Figure 2), with no changes in fGNG due to TBI (Con: 71.23% ± 5.38 vs. TBI: 69.47 ± 4.77%, *p* = 0.50). There was a similar increase in fGNG between the CR-Con (79.83 ± 4.43%) and CR+TBI (79.10 ± 4.52%) groups (*p* ≥ 0.78), both being significantly higher than the AL-Con (62.64 ± 6.33% *p* < 0.001) and AL+TBI (59.85 ± 5.01% *p* ≤ 0.001) groups (Figure 3), and there was no interaction effect between CR and TBI on fGNG. Notably, the 24-h fast imparted on all animals caused a significant increase in fGNG post-intervention in both the AL-Con (Pre: 35.6 ± 1.1% vs. Post: 62.64 ± 6.33%. *p* ≤ 0.001) and AL+TBI (Pre: 35.2 ± 1.1% vs. Post: 59.85 ± 5.01% *p* ≤ 0.001). Thus, there is a significant effect of feeding on fGNG independent of TBI. At the conclusion of the study, there were no significant differences in blood glucose concentrations with CR or TBI (AL-Con: 167.5 ± 17.9 mg/dL, AL+TBI: 176.5 ± 25.1 mg/dL, CR-Con: 162.3 ± 24.0 mg/dL, and CR+TBI: 158.0 ± 10.0mg/dL: *p* > 0.5).

## 4. Discussion

The purpose of this study was to determine the individual and combined effects of CR and TBI on fGNG. We found that fGNG was significantly impacted by CR, independent of TBI. Hence, the results indicate that following CR, energy reserves are mobilized to support blood glucose homeostasis by increasing fGNG. Further, our study shows the potential of using fGNG via D_2_O administration to assess caloric needs, particularly in individuals that are recovering from a TBI.

In nondiabetic individuals, gluconeogenic rates are known to respond to feeding, fasting, and metabolic activity as glucose reserves become depleted and must be replenished during and after periods of fasting [14]. Consequently, if dietary sources are inadequate to maintain euglycemia, gluconeogenesis is reflexively elevated, making fGNG, not blood glucose concentration, a biomarker of dietary energy adequacy [27]. Knowing, and supporting body energy requirements following injury seems prudent considering the metabolic crisis common with TBI that serves to deplete endogenous nutrient and body mass energy stores. This experiment demonstrates that 50% caloric restriction in rats is sufficient feeding stress to induce significantly elevated rates of fGNG.

### 4.1. TBI and Glucose Control

The results of the present investigation on TBI and control rats are comparable with those obtained on human patients that suffered a TBI [6]. In the Neuro ICU glucose flux was determined from a primed-continuous infusion of [6,6-D]glucose and fGNG was determined from the incorporation of ^13^C from infused [3-^13^C]lactate into plasma glucose [6,12]. TBI patients exhibited a fGNG of 67%, twofold greater than in the post-absorptive controls [6]. Retrospectively, in consideration of results obtained in the present investigation, the increase in fGNG values in post-TBI human patients may have been due to caloric restriction, not the condition of TBI. The comparison of results obtained with primed-continuous infusions of [6,6-D]glucose and a [3-^13^C]lactate on humans in a Neuro-ICU and on rats using the D_2_O method may be informative. The former, primed-continuous infusion studies were short-term [6,12], 3-h experiments, whereas the D_2_O labeled drinking water studies have the potential for long-term labeling measurements spanning days to months [22]. Thus, D_2_O labeling has the potential to measure fGNG for the duration of recovery, allowing the practitioner to adjust caloric support as patient energy needs change. It is noteworthy that instead of labeling drinking water as presented in the current study, patients could be continuously administered D_2_O water via nasal gastric feeding or by a labeled saline iv drip.

Since Banting and Best discovered insulin a century ago [28], the biomedical world has been dominated by monitoring and controlling glucose concentration. In the ICU, tight glycemic control is a priority [29,30]. Initially, TBI patients can be hyperglycemic [9], yet glucose cerebral metabolic rate (CMR) is severely decreased post-TBI [12]. With the current SOC, practitioners titrate insulin to decrease blood glucose levels, which decreases the blood glucose availability to the patient’s brain post-injury [31]. Further impacting patient recovery, underfeeding exacerbates the metabolic crisis caused by the illness or injury [32]. Post-TBI, the brain needs glucose, yet the body is vastly underfed causing an increase in endogenous glucose production via GNG; however, insulin titration suppresses the availability of the newly produced glucose exacerbating metabolic crises and further influencing fGNG.

### 4.2. Clinical Ramifications of under/over Feeding, Benefits of Using fGNG as a Biomarker

Influential practitioners in critical care nutrition stress the need for a biomarker to guide nutrition during the critical care and the anabolic phases of recovery [33]. fGNG may have particular applications in the acute phase of critical care to tailor caloric administration for non-diabetic patients and thus avoid underfeeding. Classically, healthcare professionals have used the H–B equation to predict the caloric needs of patients [34]; yet, in clinical settings, the H–B equation may vastly underestimate the caloric needs of critically ill patients, especially those post-TBI [3,4,33]. In acute care TBI patients (<30 days post-injury), resting energy expenditure has been underestimated by 87 to 200% [3]. For example, McEvoy and colleagues [4] demonstrated that predictive equations can differ up to 550 kcal from the patient’s actual energy requirements, leading to the underfeeding of patients. Such significant energy deficits lead to catabolism of lean tissue that challenges the healing process via lean body wasting [6,12].

While we have noted limitations in H–B equations for application in critical care settings, in fact, all static measurements, such as blood glucose concentration, as well as predictive equations based on regression to mean, utilized to evaluate energy needs, lack the ability to provide personalized assessments of body energy state [17,35,36]. By contrast, using a small, inexpensive dose of D_2_O, followed by mass spectrometry measurements of glucose and body water isotopic enrichments, those treating patients during recovery from illness and injury can readily and repeatedly assess the caloric needs of their patients over the course of recovery. With this information, it may be possible to make an accurate individualized caloric assessment to help maximize recovery. Importantly, by measuring fGNG from a small blood sample in an existing hospital, Clinical Laboratory Improvement Amendments (CLIA) laboratory, there is an opportunity for reimbursement using existing current procedural terminology (CPT) codes.

fGNG measurements are not only an indicator of underfeeding but can also give insight into whether the patient is overfed. Although overfeeding is not common during the acute phase of illness or injury, there is evidence to suggest that overfeeding may be detrimental to patient recovery [37]. In ICU patients, There is an increase in inflammation and blood infections with overfeeding [37]. The dynamic nature of fGNG allows medical staff to measure the fed (0~15%) or unfed state (greater than 40%) of a patient. If fGNG is continuously monitored, in non-diabetic patients on a and in the lower range (0–10%), this could indicate that the patient is overfed. By measuring fGNG healthcare practitioners will have the necessary evaluations to assess the dietary energy states of patients.

Finally, previous results showing elevated fGNG in TBI patients were likely due to underfeeding, then trauma [6,12,18]. Lactate, a major gluconeogenic precursor [38,39], has shown a vast (71%) increase in the rate of appearance in TBI subjects compared to control subjects [6]. While some of this lactate is disposed of via direct oxidation [12,18], most is converted into glucose via GNG [12]. With the mounting evidence of increased fGNG post-TBI, likely stemming from underfeeding, the *corpus* is being sacrificed to provide support for the brain post-injury.

### 4.3. Limitations

The study was designed to induce a moderate TBI mimicking a sports concussion. However, the behavioral data and decreased voluntary food consumption 24 h post-injury indicate that we administered a mild TBI [2,40]. Possibly a more severe injury may have caused an alteration in fGNG even with adequate nutrition. Despite limited sample sizes, we showed large effect sizes (*p* ≤ 0.001) and the fGNG values obtained from our control animals were similar to those in previous studies [16,41]. Another limitation of our study was the lack of macronutrient composition manipulation; rather, we used a SOC diet found in a hospital setting.

### 4.4. Future Research Direction

The results of this study may encourage healthcare providers to conduct clinical trials using fGNG as a biomarker of caloric adequacy in trauma patients. In addition to assessing body energy needs using determinations of fGNG, such studies could include simultaneous assessments of dietary energy need using traditional methods. In addition, future studies could include manipulations of macronutrient composition to assess effects on fGNG, lean body mass, and desired clinical outcomes. In these ways, assessments of fGNG by D_2_O methodology may lead to better outcomes in the care of trauma patients

## 5. Conclusions

Caloric restriction increases fractional gluconeogenesis (fGNG) independent of injury as imposed by TBI. Thus, fGNG may be a viable biomarker for the assessment of caloric adequacy during recovery

## Figures and Tables

**Table 1:** TBI and CR significantly decreased body weight 24 h post-injury.

Group	Number of Animals	Pre-Intervention	24 h Post-Intervention	13 Days Post-Intervention	Total Weight Change
AL-Con	3	249.3 ± 2.1 g	248.7 ± 0.6 g	294.3 ± 6.7 g	45.0 ± 6.2 g
AL+TBI	6	252.2 ± 15.8 g	241.2 ± 15.5 g *	285.3 ± 16.1 g	33.2 ± 20.6 g
CR-Con	6	292.16 ± 14.8 g *^+^^	291.5 ± 15.3 g	278.3 ± 10.6 g	−13.83 ± 13.2 g *^+^
CR+TBI	5	244.8 ± 13.9 g	233.0 ± 12.1 g *	216.6015.8 g *^$+^	−28.20 ± 7.0 g *^+^

Body weight changes (grams) from pre-intervention to 24 h and 13 days post-intervention, and relative body weight changes. * Significantly different from AL-Con *p* ≤ 0.05, ^+^ significantly different from AL+TBI *p* ≤ 0.05, ^$^ significantly different from CR-Con *p* ≤ 0.05, ^^^ significantly different from CR+TBI *p* ≤ 0.05.

**Table 2:** TBI and CR significantly decreased food consumption 24 h post-injury.

Group	Number of Animals	Pre-Intervention	24 Hours Post-Intervention	13 Days Post-Intervention
AL-Con	3	22.5 ± 0.8 g	18.0 ± 2.6 g	21.1 ± 1.4 g
AL+TBI	6	22.7 ± 1.2 g	12.3 ± 4.5 g *^&^	21.0 ± 1.5 g
CR Con	6	25.0 ± 1.2 g	12.8 ± 0.4 g *^&^	12.8 ± 0.4 g *^+&^
CR+TBI	5	21.9 ± 1.9 g	11.0 ± 1.0 g *^&^	11.0 ± 1.0 g *^+&^

Food consumption (grams) from pre-intervention, 24 h, and 13 days post-intervention. * Significantly different from AL-Con *p* ≤ 0.05, ^+^ significantly from AL TBI *p* ≤ 0.05, ^&^ significantly different from pre-intervention food consumption *p* ≤ 0.005.

## Data Availability

The data presented in this study are available in the main article.

## References

[1] Peterson A.B., Thomas K.E., Zhou H. (2022). Surveillance Report of Traumatic Brain Injury-related Deaths by Age Group, Sex, and Mechanism of Injury—United States, 2018 and 2019.

[2] TalavageThomas M., NaumanEric A., BreedloveEvan L., DyeAnne E., MorigakiKatherine E., LeverenzLarry J. (2014). Functionally-detected cognitive impairment in high school football players without clinically-diagnosed concussion. J. Neurotrauma.

[3] Rattanachaiwong S., Singer P. (2019). Indirect calorimetry as point of care testing. Clin. Nutr..

[4] McEvoy C.T., Cran G.W., Cooke S.R., Young I.S. (2009). Resting energy expenditure in non-ventilated, non-sedated patients recovering from serious traumatic brain injury: Comparison of prediction equations with indirect calorimetry values. Clin. Nutr..

[5] Pepe J.L., Barba C.A. (1999). The metabolic response to acute traumatic brain injury and implications for nutritional support. J. Head Trauma Rehabil..

[6] Glenn T.C., Martin N.A., McArthur D.L., Hovda D.A., Vespa P., Johnson M.L., Horning M.A., Brooks G.A. (2015). Endogenous nutritive support after traumatic brain injury: Peripheral lactate production for glucose supply via gluconeogenesis. J. Neurotrauma.

[7] Charrueau C., Belabed L., Besson V., Chaumeil J.-C., Cynober L., Moinard C. (2009). Metabolic response and nutritional support in traumatic brain injury: Evidence for resistance to renutrition. J. Neurotrauma.

[8] Moinard C., Neveux N., Royo N., Genthon C., Marchand-Verrecchia C., Plotkine M., Cynober L. (2005). Characterization of the alteration of nutritional state in brain injury induced by fluid percussion in rats. Intensive Care Med..

[9] Shi J., Dong B., Mao Y., Guan W., Cao J., Zhu R., Wang S. (2016). Traumatic brain injury and hyperglycemia, a potentially modifiable risk factor. Oncotarget.

[10] Maxwell J., Gwardschaladse C., Lombardo G., Petrone P., Policastro A., Karev D., Prabhakaran K., Betancourt A., Marini C. (2017). The impact of measurement of respiratory quotient by indirect calorimetry on the achievement of nitrogen balance in patients with severe traumatic brain injury. Eur. J. Trauma Emerg. Surg..

[11] Dickerson R.N., Pitts S.L., Maish III G.O., Schroeppel T.J., Magnotti L.J., Croce M.A., Minard G., Brown R.O. (2012). A reappraisal of nitrogen requirements for patients with critical illness and trauma. J. Trauma Acute Care Surg..

[12] Glenn T.C., Martin N.A., Horning M.A., McArthur D.L., Hovda D.A., Vespa P., Brooks G.A. (2015). Lactate: Brain fuel in human traumatic brain injury: A comparison with normal healthy control subjects. J. Neurotrauma.

[13] Vespa P., Bergsneider M., Hattori N., Wu H.-M., Huang S.-C., Martin N.A., Glenn T.C., McArthur D.L., Hovda D.A. (2005). Metabolic crisis without brain ischemia is common after traumatic brain injury: A combined microdialysis and positron emission tomography study. J. Cereb. Blood Flow Metab..

[14] Allick G., van der Crabben S.N., Ackermans M.T., Endert E., Sauerwein H.P. (2006). Measurement of gluconeogenesis by deuterated water: The effect of equilibration time and fasting period. Am. J. Physiol.-Endocrinol. Metab..

[15] Chung S.T., Chacko S.K., Sunehag A.L., Haymond M.W. (2015). Measurements of gluconeogenesis and glycogenolysis: A methodological review. Diabetes.

[16] Hellerstein M.K., Neese R., Linfoot P., Christiansen M., Turner S., Letscher A. (1997). Hepatic gluconeogenic fluxes and glycogen turnover during fasting in humans. A stable isotope study. J. Clin. Investig..

[17] Stoppe C., Wendt S., Mehta N.M., Compher C., Preiser J.-C., Heyland D.K., Kristof A.S. (2020). Biomarkers in critical care nutrition. Crit. Care.

[18] Glenn T.C., Kelly D.F., Boscardin W.J., McArthur D.L., Vespa P., Oertel M., Hovda D.A., Bergsneider M., Hillered L., Martin N.A. (2003). Energy dysfunction as a predictor of outcome after moderate or severe head injury: Indices of oxygen, glucose, and lactate metabolism. J. Cereb. Blood Flow Metab. Off. J. Int. Soc. Cereb. Blood Flow Metab..

[19] Brooks G.A., Martin N.A. (2015). Cerebral metabolism following traumatic brain injury: New discoveries with implications for treatment. Front. Neurosci..

[20] Orendorff R., Peck A.J., Zheng B., Shirazi S.N., Ferguson R.M., Khandhar A.P., Kemp S.J., Goodwill P., Krishnan K.M., Brooks G.A. (2017). First in vivo traumatic brain injury imaging via magnetic particle imaging. Phys. Med. Biol..

[21] Council N. (1995). Nutrient requirements of laboratory animals. The National Academies.

[22] Miller B.F., Reid J.J., Price J.C., Lin H.-J.L., Atherton P.J., Smith K. (2020). CORP: The use of deuterated water for the measurement of protein synthesis. J. Appl. Physiol..

[23] Abbott C.B., Lawrence M.M., Kobak K.A., Lopes E.B.P., Peelor F.F., Donald E.J., Van Remmen H., Griffin T.M., Miller B.F. (2021). A novel stable isotope approach demonstrates surprising degree of age-related decline in skeletal muscle collagen proteostasis. Function.

[24] Chacko S.K., Sunehag A.L., Sharma S., Sauer P.J., Haymond M.W. (2008). Measurement of gluconeogenesis using glucose fragments and mass spectrometry after ingestion of deuterium oxide. J. Appl. Physiol..

[25] Tagge C.A. (2016). Effects of Concussive Impact Injury Assessed in a New Murine Neurotrauma Model. Doctoral Dissertation.

[26] Thiels E., Hoffman E.K., Gorin M.B. (2008). A reliable behavioral assay for the assessment of sustained photophobia in mice. Curr. Eye Res..

[27] Brooks G.A. (2020). The precious few grams of glucose during exercise. Int. J. Mol. Sci..

[28] Banting F.G., Best C.H., Collip J.B., Campbell W.R., Fletcher A.A. (1922). Pancreatic extracts in the treatment of diabetes mellitus. Can. Med. Assoc. J..

[29] Van den Berghe G., Wouters P., Weekers F., Verwaest C., Bruyninckx F., Schetz M., Vlasselaers D., Ferdinande P., Lauwers P., Bouillon R. (2001). Intensive Insulin Therapy in Critically Ill Patients. N. Engl. J. Med..

[30] Investigators N.-S.S. (2009). Intensive versus conventional glucose control in critically ill patients. N. Engl. J. Med..

[31] Oddo M., Schmidt J.M., Carrera E., Badjatia N., Connolly E.S., Presciutti M., Ostapkovich N.D., Levine J.M., Le Roux P., Mayer S.A. (2008). Impact of tight glycemic control on cerebral glucose metabolism after severe brain injury: A microdialysis study. Crit. Care Med..

[32] Dhandapani S., Dhandapani M., Agarwal M., Chutani A.M., Subbiah V., Sharma B.S., Mahapatra A.K. (2012). The prognostic significance of the timing of total enteral feeding in traumatic brain injury. Surg. Neurol. Int..

[33] Casaer M.P., Van den Berghe G. (2014). Nutrition in the acute phase of critical illness. N. Engl. J. Med..

[34] Harris J.A., Benedict F.G. (1918). A biometric study of human basal metabolism. Proc. Natl. Acad. Sci. USA.

[35] Bharadwaj S., Ginoya S., Tandon P., Gohel T.D., Guirguis J., Vallabh H., Jevenn A., Hanouneh I. (2016). Malnutrition: Laboratory markers vs nutritional assessment. Gastroenterol. Rep..

[36] Parent B., Seaton M., O’Keefe G.E. (2018). Biochemical markers of nutrition support in critically ill trauma victims. J. Parenter. Enter. Nutr..

[37] Griffiths R.D. (2007). Too much of a good thing: The curse of overfeeding. Crit. Care.

[38] Gerich J.E., Meyer C., Woerle H.J., Stumvoll M. (2001). Renal gluconeogenesis: Its importance in human glucose homeostasis. Diabetes Care.

[39] Bergman B.C., Horning M.A., Casazza G.A., Wolfel E.E., Butterfield G.E., Brooks G.A. (2000). Endurance training increases gluconeogenesis during rest and exercise in men. Am. J. Physiol.-Endocrinol. Metab..

[40] Callahan M.L., Lim M.M. (2018). Sensory sensitivity in TBI: Implications for chronic disability. Curr. Neurol. Neurosci. Rep..

[41] Tayek J.A., Katz J. (1996). Glucose production, recycling, and gluconeogenesis in normals and diabetics: A mass isotopomer [U-13C] glucose study. Am. J. Physiol.-Endocrinol. Metab..

